# Management of a complex central bile duct injury associated with laparoscopic cholecystectomy: a case report of double hepaticojejunostomy and advanced transhepatic interventional drainage technique

**DOI:** 10.1093/jscr/rjaf726

**Published:** 2025-09-08

**Authors:** Michelle Ordieres, Lukas Beyer, Julia Möller, Frank Marusch, Sven Christian Schmidt

**Affiliations:** Department of Hepato-Pancreato-Biliary Surgery, Clinic for General, Visceral and Vascular Surgery, Ernst von Bergmann Klinikum, Charlottenstraße 72, 14467 Potsdam, Germany; HMU, Health and Medical University, Olympischer Weg 1, 14471 Potsdam, Germany; HMU, Health and Medical University, Olympischer Weg 1, 14471 Potsdam, Germany; Department of Diagnostic and Interventional Radiology, Ernst von Bergmann Klinikum, Charlottenstraße 72, 14467 Potsdam, Germany; Department of Hepato-Pancreato-Biliary Surgery, Clinic for General, Visceral and Vascular Surgery, Ernst von Bergmann Klinikum, Charlottenstraße 72, 14467 Potsdam, Germany; HMU, Health and Medical University, Olympischer Weg 1, 14471 Potsdam, Germany; Department of Hepato-Pancreato-Biliary Surgery, Clinic for General, Visceral and Vascular Surgery, Ernst von Bergmann Klinikum, Charlottenstraße 72, 14467 Potsdam, Germany; HMU, Health and Medical University, Olympischer Weg 1, 14471 Potsdam, Germany; Department of Hepato-Pancreato-Biliary Surgery, Clinic for General, Visceral and Vascular Surgery, Ernst von Bergmann Klinikum, Charlottenstraße 72, 14467 Potsdam, Germany; HMU, Health and Medical University, Olympischer Weg 1, 14471 Potsdam, Germany

**Keywords:** complex bile duct injury, cholecystectomy, bile duct injury repair, hepaticojejunostomy

## Abstract

We describe a case of a 64-year-old obese woman with a history of severe acute cholecystitis and choledocholithias who underwent laparoscopic cholecystectomy in our clinic after endoscopic treatment by sphincterotomy and stent insertion. On the first operative day, a significant bile leakage of 400 ml appeared in the drainage. An immediate surgical revision was performed, starting by laparoscopy with conversion to open surgery. Three separate bile ducts just above the level of the biliary confluence were revealed intraoperatively. The injury was addressed by performing two separate hepaticojejunostomy anastomoses, supported by transanastomotic drains to the left sided ducts. Additionally, a percutaneous transhepatic cholangiography drain was inserted in the right hepatic duct. The postoperative course was uneventful and at postoperative day 16 the patient was discharged at home. After a follow-up of 18 months, the patient is in good condition without any signs of biliary complications. This case highlights the challenge of managing complex bile duct injuries. It emphasizes the need for multidisciplinary management in a dedicated hepato-biliary center with experienced surgeons, endoscopists, and interventional radiologists.

## Introduction

Laparoscopic cholecystectomy carries a higher risk of major bile duct injury (BDI) compared to open surgery, with reported rates of 0.4%–1.5% after laparoscopic cholecystectomy vs. 0.2%–0.3% following open surgery [1]. Minor BDI can be managed effectively by endoscopic techniques such as endoscopic retrograde cholangiopancreatography (ERCP) with success rates ranging from 87% to 100% [2]. Conversely, major BDIs are typically associated with significant tissue loss, necessitating surgical treatment.

Herewith, we present a case of a severe bile duct injury occurred in our clinic. Early detected by postoperative high volume biliary fistula, the patient was re-operated and definitively reconstructed by a double hepaticojejunostomy (HJ) at postoperative day 1. The management of major bile duct injury associated with cholecystectomy is described and discussed.

## Case presentation

**Figure 1:**
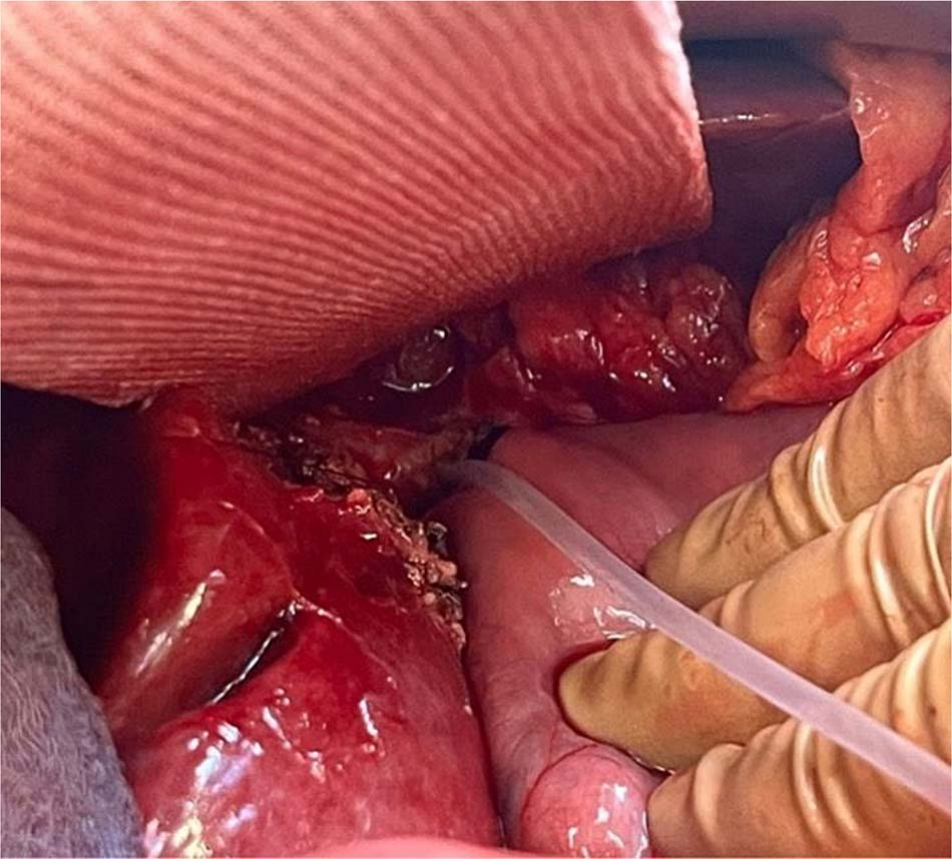
Conjunction of the left-sided bile ducts into a single hepaticojejunostomy. The right-sided duct required a separate hepaticojejunostomy using the same jejunal limb. To prevent complications, transhepatic external-internal biliary drains were placed in the left-sided anastomosis, extending into the jejunum.

A 64-year-old obese woman presented to the emergency department with clinical signs of cholestasis. Initial imaging identified cholecystolithiasis and bile duct dilatation, later confirmed as choledocholithiasis visualized on endoscopic ultrasound. Subsequently, the patient underwent ERCP with papillotomy, stone removal and stent placement. Seven weeks later, an elective laparoscopic cholecystectomy was performed. Severe inflammation of the surrounding tissue was noted intraoperatively. The gallbladder was shrunken and embedded within the liver parenchyma, hindering the critical view of safety. On the first postoperative day, significant bile leakage of 400 ml was observed prompting an immediate exploratory laparoscopic revision by our hepatobiliary team. Here, a transection of the extrahepatic bile duct just above the hepatic duct confluence was detected, resulting in three separate ostia: two on the left side (left main duct and segment IV bile duct) and main right hepatic duct. To reconstruct the biliary anatomy the procedure was converted to open surgery. The two left-sided bile ducts were joined together by two interrupted stiches to create a common channel for the Roux-Y-hepaticojejunostomy. The right-sided duct required a separate HJ 3 cm proximally on the same jejunal limb. To prevent bile leakage and stenosis two external-internal biliary drains were placed in the left-sided anastomosis, routed transhepatically and ending in the jejunum (Fig. 1). Due to technical challenges related to the patient’s obesity a third external-internal biliary drain could not be placed in the right-sided HJ. The biliodigestive anastomosis was constructed with an interrupted absorbable 5/0 monofil sutures in duct-to extra mucosal technique.

**Figure 2:**
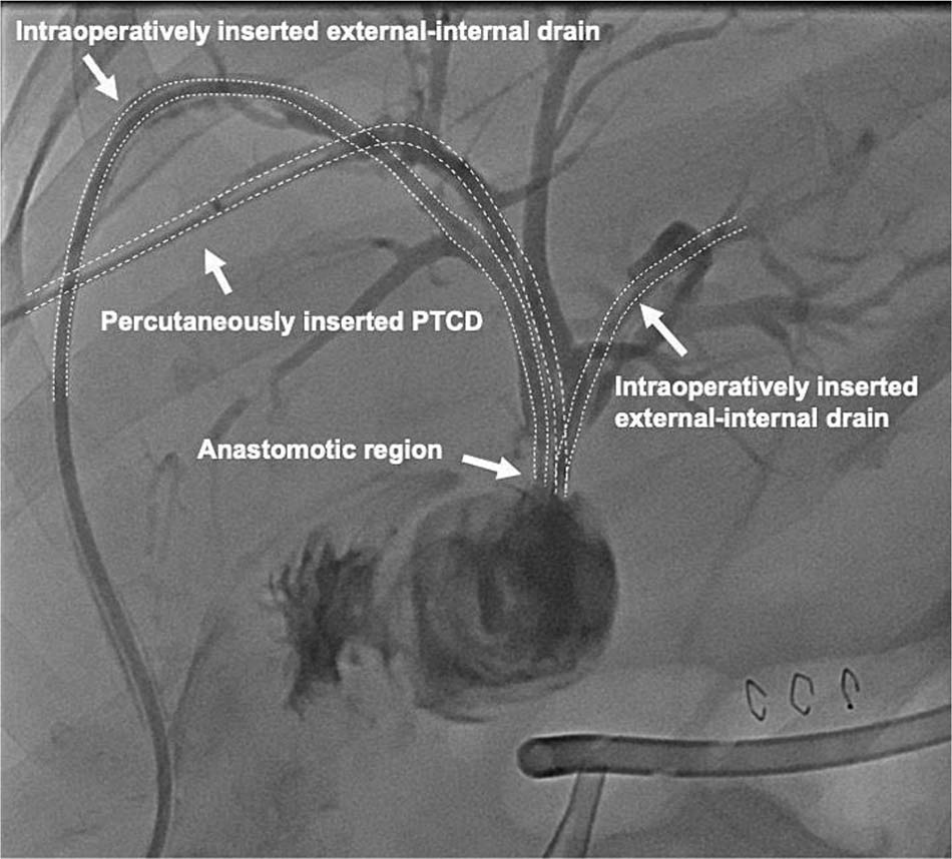
Contrast-enhanced image of the intraoperatively inserted external-internal drains and the percutaneous transhepatic cholangiography drainage (PTCD) which was interventionally inserted into the right duct on postoperative day one.

**Figure 3:**
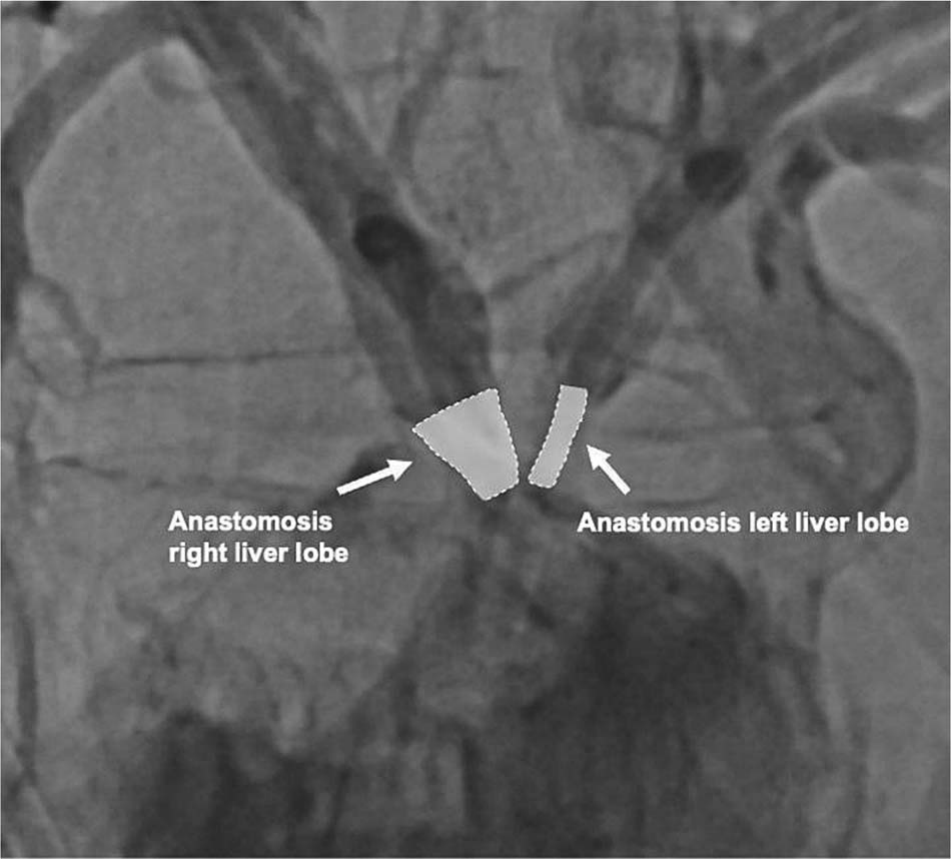
Contrast-enhanced image of both hepaticojejunostomy anastomoses 4 weeks after biliary reconstruction.

On the following day, a percutaneous transhepatic cholangiography drainage (PTCD) was placed in the right duct via interventional radiology for additional bile drainage support (Fig. 2). The patient was discharged on postoperative day 16 with the three closed drains in place. Four weeks later, she was readmitted for drain removal, with imaging (Fig. 3) and laboratory results (total bilirubin 8.4 μmol/L (norm <17 μmol/L) showing no signs of stenosis or leakage. After a follow-up of 18 months, the patient is in good condition with no clinical signs of late biliary complications.

## Discussion

BDI remains a significant complication of laparoscopic cholecystectomy. BDI may result in significant morbidity and mortality [3] and also have a considerable negative impact on long-term quality-of-life [4]. Once a postoperative bile leak is suspected, accurate diagnostic imaging by contrast enhanced multi-phase abdominal computed tomography followed by magnetic resonance imaging is crucial to describe exactly the extent of the lesion. While minor BDIs can usually be treated by endoscopic options with a high success rate of over 90% [5], major BDIs mostly require surgical treatment. The surgical options range from simple suture repair of the bile duct to more sophisticated procedures such as HJ, in some cases combined with vascular reconstruction, up to liver resection [6].

A matter of debate is the optimal timing of repair. In the current literature an early repair is generally not recommended before the patient is not stable and in good condition with resolved bile leak by interventional treatment [4]. The rational for a delayed definitive repair is the high postoperative morbidity associated in patients operated in existing sepsis and biliary peritonitis [7]. Otherwise, in cases with intraoperative diagnosed major biliary injury, the patient should be either operated immediately by an experienced hepatobiliary surgeon or, if not possible, referred to a tertiary center. Here we present a patient with a major BDI with a transection of the hepatic duct confluence. We decided to re-laparoscopy this patient due to the huge amount of bile fluid in the drainage. After detection of the leak, the operation was converted to open procedure. In this case we avoided intraoperative cholangiography due to clear identification of the biliary anatomy. Biliary reconstruction by Roux-en-Y HJ is the preferred method of repair of severe BDI [8]. Generally the HJ should be performed at a high level due to a better blood supply of the bile duct and thus to prevent later ischemic strictures [9]. The HJ can be performed as end-to-side as well as side-to-side anastomosis incorporating the hepatic confluence either with the right or left hepatic duct providing a wide anastomosis. As in our case, in high injuries two bile duct stumps can be joined to create a wider biliary ostium and if necessary two or more hepaticojejunal anastomoses can be created [8]. Reports of multiple anastomoses for separated bile ducts are relatively scarce. In our case, a double HJ accompanied by the placement of external-internal drain and postoperative PTCD was successfully performed. Though, limited literature exists on intraoperative stenting to prevent anastomotic insufficiency or stenosis. Treated in experienced centers the long-term outcome after bile duct reconstruction by HJ is reported between 80% and 90% [10]. However anastomotic stricture with subsequent recurrent cholangitis up to development of secondary biliary cirrhosis are late complications. Thus, it is necessary to evaluate these patients regularly in the follow-up period to detect and treat these complications early.

## Conclusion

This case report highlights the critical importance of early recognition of bile duct injury. The case underscores the efficacy of a multidisciplinary team. Rapid patient transfer to specialized centers should be prioritized to optimize care and achieve the best possible results.
